# TeCD: The eccDNA Collection Database for extrachromosomal circular DNA

**DOI:** 10.1186/s12864-023-09135-5

**Published:** 2023-01-27

**Authors:** Jing Guo, Ze Zhang, Qingcui Li, Xiao Chang, Xiaoping Liu

**Affiliations:** 1 Key Laboratory of Systems Biology, Hangzhou Institute for Advanced Study, University of Chinese Academy of Sciences, Hangzhou, 310013 China; 2 Key Laboratory of Systems Health Science of Zhejiang Province, Hangzhou Institute for Advanced Study, University of Chinese Academy of Sciences, Hangzhou, 310013 China; 3 Institute of Statistics and Applied Mathematics, Anhui University of Finance & Economics, Bengbu, 233030 China; 4 School of Mathematics and Statistics, Shandong University, Weihai, 264209 Shandong China; 5 School of Life Science, Hangzhou Institute for Advanced Study, University of Chinese Academy of Sciences, Hangzhou, 310013 China

**Keywords:** Circular DNA, Extrachromosomal DNA, eccDNA

## Abstract

**Background:**

Extrachromosomal circular DNA (eccDNA) is a kind of DNA that widely exists in eukaryotic cells. Studies in recent years have shown that eccDNA is often enriched during tumors and aging, and participates in the development of cell physiological activities in a special way, so people have paid more and more attention to the eccDNA, and it has also become a critical new topic in modern biological research.

**Description:**

We built a database to collect eccDNA, including animals, plants and fungi, and provide researchers with an eccDNA retrieval platform. The collected eccDNAs were processed in a uniform format and classified according to the species to which it belongs and the chromosome of the source. Each eccDNA record contained sequence length, start and end sites on the corresponding chromosome, order of the bases, genomic elements such as genes and transposons, and other information in the respective sequencing experiment. All the data were stored into the TeCD (The eccDNA Collection Database) and the BLAST (Basic Local Alignment Search Tool) sequence alignment function was also added into the database for analyzing the potential eccDNA sequences.

**Conclusion:**

We built TeCD, a platform for users to search and obtain eccDNA data, and analyzed the possible potential functions of eccDNA. These findings may provide a basis and direction for researchers to further explore the biological significance of eccDNA in the future.

## Background

In eukaryotes, a particular class of circular DNA molecules is separated from the normal genome, dissociated from the chromosome, and participates in physiological or pathological processes with a special way [1]. Because these molecules are independent DNA molecules outside the chromosome, and, they are often circular, so they are called extrachromosomal circular DNA, or eccDNA [2]. 

A lot of studies have shown that eccDNA can be involved in a wide range of biological processes [3–5]. For example, in cancer, eccDNA is the main factor leading to tumor heterogeneity and is regarded as a marker of genomic instability. However, eccDNA is also detected in healthy cells and can appear in a tissue-specific or developmentally regulated mode, which implies possible physiological effects [3]. Although researchers have found a large amount of eccDNA in normal and cancer cells [6, 7], people are still paying more and more attention to the connection between eccDNA and cancer [8–10]. Many studies have shown that eccDNA plays an important role in genetic variation, evolution, genomic instability, genomic plasticity, drug resistance, environmental adaptation, mutation and tumorigenesis [8]. An eccDNA usually contains tumor genes or drug resistance genes [9–13], so it can promote cancer cells to gain growth or survival advantages, and it can also lead to gene deletion, mutation, duplication, amplification or migration, intercellular genetic heterogeneity, and adaptive evolution [9]. Through the study of colon cancer HT29 cells, ovarian cancer, and breast cancer tissues, researchers found that eccDNA may promote the drug resistance of cancer or tumor cells by amplifying tumor genes [10]. One characteristic of the plasticity of the genome is the presence of eccDNA [12], which is the ability of the genome to produce different phenotypes based on different environmental cues and is related to a variety of human diseases and related phenotypes. High levels of eccDNA are related to genome instability, exposure to carcinogens, and cell aging [11]. The study of Fanconi’s anemia in DNA repair deficiency syndrome has shown genetic instability and increased levels of associated eccDNA molecules [13]. In summary, researchers are still exploring and studying the functions of eccDNA, and there is still a lot of unknown information that they have not grasped. The research of eccDNA may have a certain impact on traditional genetics and genomics in the future. 

However, the integrative resources that already exist for eccDNA, such as eccDNAdb [14] and CircleBase [15], contain only human data or specifically human tumor data as far as we know. We would like to build a database platform containing eccDNA from a variety of eukaryotes. Thus, we established a database named TeCD (The eccDNA Collection Database) to integrate the eccDNA data scattered in public literature. The species distribution of the data in TeCD covers animals, plants and fungi. Researchers can obtain the genomic elements or locations of eccDNA and detect the related sequences by BLAST (Basic Local Alignment Search Tool) from TeCD. 

## Construction and content

### Database implementation

**Fig. 1:**
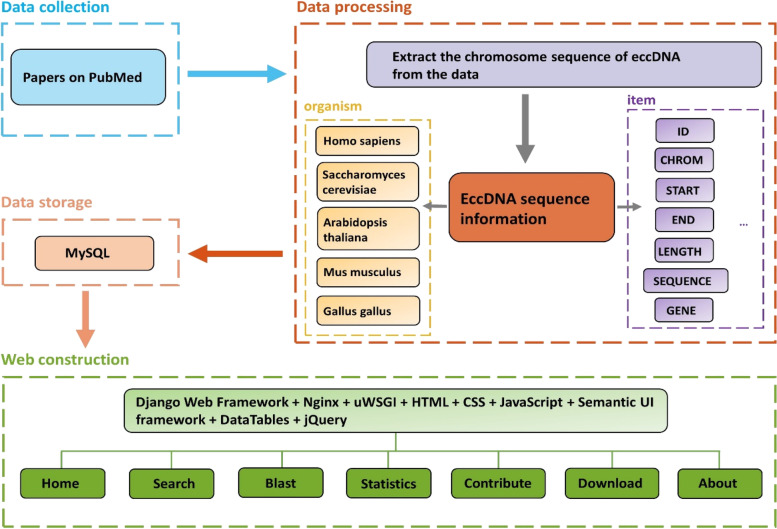
The flow chart of TeCD. The raw data were collected from published literature. The chromosome sequence corresponding to eccDNA was obtained according to the locus. The gene, locus information, number of reads variants, and other information were extracted from the data and stored in MySQL 8.0.26 database. The information could be browsed and retrieved according to organism and item. Finally, the TeCD website was built with Django web framework for users

The web server was deployed based on Nginx 1.18.0 (http://nginx.org/) and uWSGI 2.0.19.1 (https://pypi.org/project/uWSGI/), and the Django 2.2.12 framework (https://www.djangoproject.com/) was used to implement Python language interface on a back-end server. And all data were stored in MySQL 8.0.26 database ( https://dev.mysql.com/) for Linux on x86_64 (Ubuntu Server 20.04.2 LTS). The web page templates were used on Semantic UI framework (https://semantic-ui.com/), DataTables (http://datatables.net), and jQuery (http://jquery.com) to establish a user-friendly front-end interface. HTML, CSS, and JavaScript were selected as the client-side languages for front-end design (Fig. 1).

### Data collection

Many studies have proven that eccDNA comes from the chromosome, not from any pre-existing extrachromosomal precursors [16, 17], so each detected eccDNA can be matched to a corresponding site on chromosome. At present, most the studies about eccDNA only focus on both the main characteristics of eccDNA (such as length, structure, and expressive characteristics) and the development of isolating methods that can be used to detect eccDNA [18, 19]. The research for the function of eccDNA is still few. To facilitate the further exploration of the function of eccDNA, we collected the existing information of eccDNA and built a database to organize them for acquirement.

The eccDNA data were collected from the published literature related to eccDNA for eukaryotes [7, 17, 20–35], and included the information such as eccDNA type, length, locus shedding from chromosomes, sequencing approach and so on. These data are relatively scattered in different eccDNA studies and need further process after the collection of the data (Fig. 1).

### Data processing

There were about 2,343,000 eccDNAs to be collected from public literature. The eccDNAs were filtered according to whether they contained the start and end sites of eccDNA on the corresponding chromosome, and a total of 1,846,905 eccDNAs were obtained. We matched the eccDNAs through their start and end sites to the reference genome (Homo sapiens GRCh38.p13, Saccharomyces cerevisiae S288C, Arabidopsis thaliana TAIR10.1, Gallus gallus bGalGal1.mat.broiler. GRCg7b and Mus musculus GRCm39) from the National Center for Biotechnology Information (NCBI, https://www.ncbi.nlm.nih.gov/). Then, the filtered data were divided into groups according to species (Table 1). Then the repeated sequences were identified by BLAST tool [36] with a threshold of e-value ${10}^{-6}$ in individual species, and were removed from the database. After the filter, we obtained a total of 948,469 eccDNAs. The amount of eccDNA for each species is shown in Table 1.

**Table 1:** The amount of eccDNA for each organism

	Homo sapiens	Saccharomyces cerevisiae	Arabidopsis thaliana	Gallus gallus	Mus musculus
Obtained data	379,148	2111	702	1,087,503	377,441
After BLAST	193,294	1266	591	433,993	319,325

In this database, we assigned a unique identification ID which is composed of species information and alphanumeric ordering to each eccDNA. For example, ecc_Hs_100000001 is the ID of eccDNA from Homo sapiens (where ‘Hs’ represents Homo sapiens), and ecc_Sc_100000001 is the ID of eccDNA from Saccharomyces cerevisiae (where ‘Sc’ represents Saccharomyces cerevisiae). Besides, we also marked the source of each eccDNA based on the tissue and cell type of the sample, and statistically showed that there is a total of 42 categories for the source of tissue and cell type in the database. In addition, each eccDNA was marked the potential source of chromosomes and start/end sites in the chromosome. These classifications can help any scientific researcher to accurately retrieve information on the TeCD platform and carry out research work.

Next, each eccDNA was accurately matched to the corresponding genes or partially covered genes by searching the NCBI genome database. In order to facilitate scientific researchers for obtaining relevant gene information instantly when browsing and retrieving eccDNA, we have made a direct link to each matched gene, and NCBI’s gene bank will provide a comprehensive and authoritative explanation for the gene (Fig. 1). Specially, the Arabidopsis gene models are linked to TAIR (The Arabidopsis Information Resource https://www.arabidopsis.org/), the most authoritative Arabidopsis genome database and Arabidopsis genome annotation system in the world [37, 38]. Besides, the transposable elements information of the sequence is also included in TeCD.

### Classification and statistics of eccDNA

**Fig. 2:**
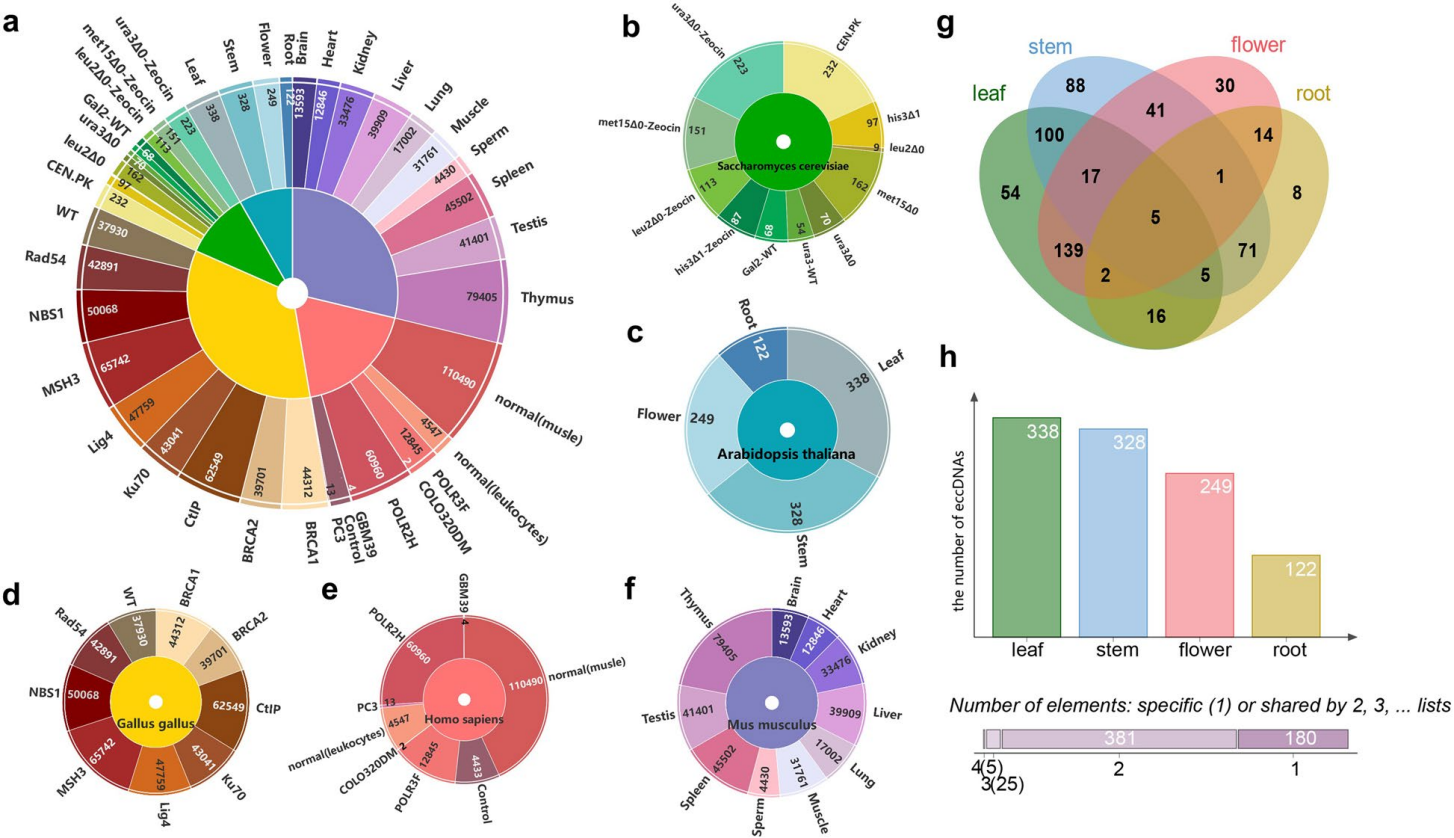
Sample types for different tissues and cells. **a** Sample types for all organisms and the corresponding number of eccDNA. The 5 colors of the inner ring correspond to 5 organisms. The outer ring represents the sample type for each organism. **b-f** Sample types of Saccharomyces cerevisiae, Arabidopsis thaliana, Gallus gallus, homo sapiens, and Mus musculus. And the color of its inner ring corresponds to the inner ring of the (**a**). **g** The number of eccDNA shared by samples from different parts of Arabidopsis. The different colored circles represent samples of different parts of Arabidopsis. **h** The figure above shows the number of eccDNA from four samples of Arabidopsis. The figure below shows the number of eccDNA that belongs to 1–4 different samples in common

The tissue and cell type to which the sample belongs is an important indicator of eccDNA classification, according to which 948,469 eccDNA records in TeCD could be summarized into 42 types (Fig. 2a). And Fig. 2b-e show sample types for five organisms. The inner circle color of each figure corresponds to the inner circle color of Fig. 2a. Samples from Saccharomyces cerevisiae are from 11 categories which consisted of the CEN.PK strain and the S288C strain, and we named samples which from CEN.PK strains as ‘CEN.PK’ and further classified samples which from the S288C strain [21, 23]. In the S288C Saccharomyces cerevisiae (Fig. 2b), ‘*MATa Gal2* isogenic WT population’ and ‘*MATa ura3* isogenic WT population’ are two wild-type Saccharomyces cerevisiae respectively and are in contrast to ‘*MATa his3Δ1* gene deletion yeast’, ‘*MATa leu2Δ0* gene deletion yeast’, ‘*MATa met15Δ0* gene deletion yeast’ and ‘*MATa ura3Δ0* gene deletion yeast’. ‘*MATa his3Δ1* gene deletion yeast’, ‘*MATa leu2Δ0* gene deletion yeast’, ‘*MATa met15Δ0* gene deletion yeast’ and ‘*MATa ura3Δ0* gene deletion yeast’ are single-gene deletion mutants without *his3Δ1*, *leu2Δ0*, *met15Δ0* and *ura3Δ0*. ‘*MATa his3Δ1* gene deletion with Zeocin’, ‘*MATa leu2Δ0* gene deletion with Zeocin’, ‘*MATa met15Δ0* gene deletion with Zeocin’ and ‘*MATa ura3Δ0* gene deletion with Zeocin’ are the samples in which DNA-damaging agent Zeocin is added to the corresponding single-gene deletion mutants [23]. *MATa* is a mating type of Saccharomyces cerevisiae [39]. The samples from Arabidopsis thaliana contain 4 categories [34], which are leaf, stem, flower, root (Fig. 2c). Notably, each eccDNA of Arabidopsis thaliana was detected from one or more samples. We intersected the number of eccDNA detected in different sample types and performed a Wayne diagram analysis of the results (Fig. 2g). The different colored circles represent the samples of different parts of Arabidopsis. As shown in Fig.2 h, 381 eccDNAs were detected in 2 samples, 25 eccDNAs were detected in 3 samples, and 5 eccDNA were detected in 4 samples. The samples from Gallus gallus contain 9 categories (Fig. 2d). These samples were derived from mutants of the DT-40 cell line [35]. For example, ‘BRCA1’ means DT40-Chicken cell line-mutated BRCA1. Samples from Homo sapiens come from 8 categories(Fig. 2e), of which ‘muscle’ represents human samples from healthy human muscle tissue, ‘leukocytes’ means leukocytes collected after centrifugation from the venous blood (40 ml) of healthy human arm veins [20]. The human embryonic kidney (HEK293) cells are easily transfected and ‘HEK293’ is the control of ‘HEK293 (+ POLR2H)’ and ‘HEK293 (+ POLR3F)’. ‘HEK293 (+ POLR2H)’ means that the POLR2H (RNA Polymerase II, I And III Subunit H) protein-coding gene was transfected into HEK293 cells and ‘HEK293 (+ POLR3F)’ means that the POLR3F (RNA Polymerase III Subunit F) protein-coding gene was transfected into HEK293 cells [22]. There is also human prostate cancer cell line ‘PC3’, colon cancer cell line ‘COLO320DM’, and human glioblastoma ‘GBM39’ tumor spheroids derived from patient tissues [24]. The samples from Mus musculus contain 10 categories (Fig. 2d). All samples are from organs or tissues of 6-months-old adult mice, including brain, heart, kidney, liver, lung, muscle, sperm, spleen, testis and thymus [35].

**Fig. 3:**
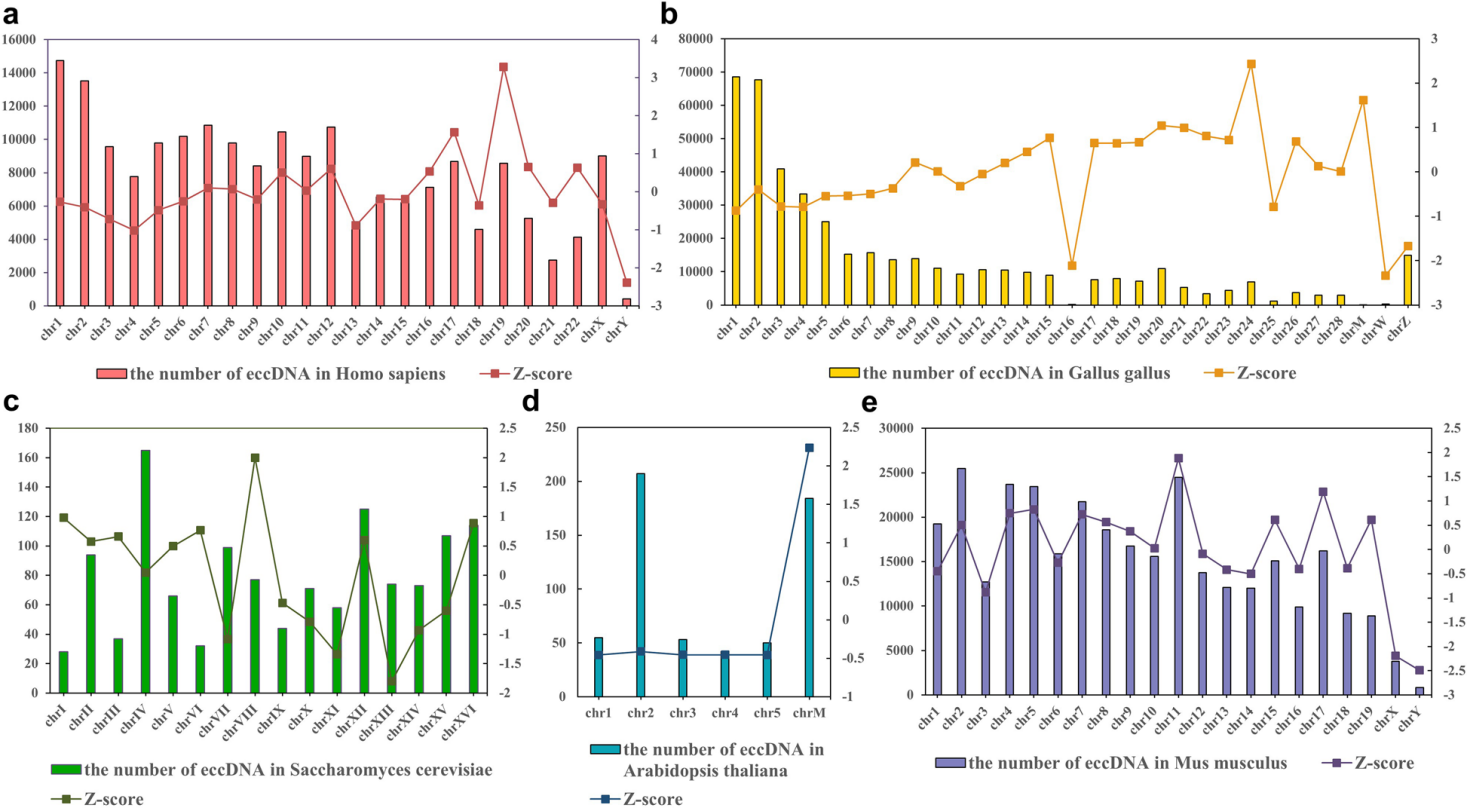
Data distribution plot. **a-e** The histogram shows the quantitative distribution of eccDNA of different organisms on chromosomes. These species are Homo sapiens, Gallus gallus, Saccharomyces cerevisiae, Arabidopsis thaliana and Mus musculus in turn. The line chart shows the ratio standardized with Z-score. And this ratio is the number of eccDNA in the chromosome divided by the length of the chromosome

Additionally, we recorded the quantitative distribution of eccDNA on chromosomes, where the histogram represents the specific amount of eccDNA on different chromosomes and the line chart represents the standardized data (Fig. 3). As each chromosome has a different length, in order to eliminate the comparative baseline heterogeneity caused by chromosome heterogeneity, we calculated the ratio of the number of eccDNA in chromosomes to the length of chromosomes and standardized the ratio by Z-score to compare the amount of eccDNA formed by shedding from different chromosomes. The standardized results were plotted into a line chart. The source of eccDNA is dispersed to 22 autosomes, 2 sexual chromosomes, and mitochondria in humans, and the amount of eccDNA from chromosomes 1, 2, 7, 12 are obviously more than other chromosomes. The eccDNA from X chromosome significantly exceeds the eccDNA from Y chromosome (Fig. 3a). From the line chart of human data, it is not difficult to find that the amount of eccDNA on chromosomes 17 and 19 accounts for the largest proportion (Fig. 3a). The genome of Gallus gallus has 39 autosomes and two sex chromosomes, but in the collected data, eccDNA only comes from 28 autosomes and 2 sex chromosomes (Fig. 3b). Mostly, the number of eccDNA on each chromosome of Saccharomyces cerevisiae was 50–150. It was found on the eighth chromosome *"ChrVIII"* of Saccharomyces cerevisiae, the ratio of eccDNA to chromosome length was the highest, reaching 0.13‰ (Fig. 3c). Most of the eccDNA of Arabidopsis thaliana comes from chromosome 2 and mitochondria. Although the length of mitochondria is the shortest, the eccDNA produced by shedding from mitochondria accounts for about one-third of the total (Fig. 3d). The eccDNA of Mus musculus is mainly derived from autosomes. Through comparison, it is not difficult to find that the source distribution of eccDNA on chromosomes of different species is diverse.

## Utility and discussion

### Web interface

To ensure that users can use these datasets of eccDNA which we collected and processed, we have developed an online website for browsing and querying the information. The whole website is divided into home, search, blast, statistics, contribute, download, and about.

#### Search

**Fig. 4:**
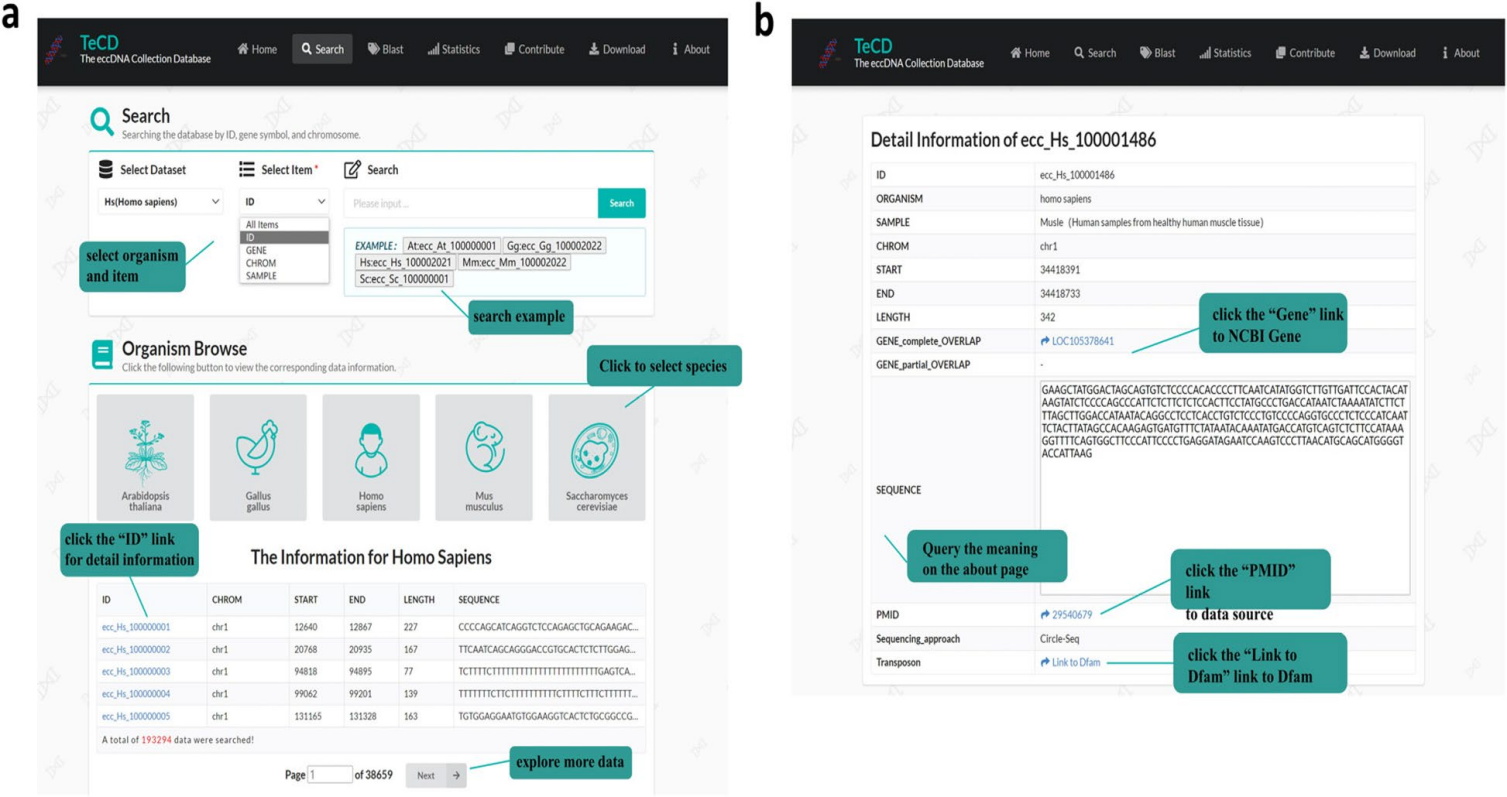
Screenshot of search page from the TeCD. **a** Search and browse pages. Users can search by organization and item, and we also provide some examples. Selected entry information will be displayed at the bottom of the page for users to browse. **b** Details of an eccDNA. Here, we provide a lot of information about eccDNA, including complete sequence, contained genes, transposons, data sources, etc. All information categories can be queried and explained on the about page. **c** JBrowse style visualization of individual eccDNAs and their location on the host genome

On the search page, a user can search and browse the basic information sets of eccDNA molecule (Fig. 4a and b). Users can select species or enter ID, gene, chromosome and sample type to retrieve eccDNA directionally. The retrieved eccDNA will be displayed at the bottom of the page. We have also placed examples for users’ reference by class under the input box of retrieval. Of course, users can directly view the information of eccDNAs related to a species by clicking the button in “Organism Browse”, and the information includes the ID of the eccDNA in the TeCD database, the chromosome to which it belongs, start and end sites in the chromosome, length of the sequence, nucleotide arrangement of the sequence (Fig. 4a). By clicking the ID of the eccDNA, it would jump to a detailed information page of the eccDNA. The detail page provides more information about the eccDNA (Fig. 4b). Users can find the notes of each row of information on the “About” page.

The information of genes covered by a retrieval eccDNA can be obtained by clicking the gene symbol and jumping to detailed page of gene in NCBI, and detailed function of the gene can be shown in the NCBI page. Each transposable element of eccDNA is linked to a visualization page of Dfam (https://www.dfam.org/) which is a eukaryotic transposable element database [40, 41]. It can visualize the distribution of transposable subcomponents on a sequence, provide a detailed interpretation of components and download the data. And the retrieval data of Dfam is highly matched with the data in TeCD such as eccDNA sites. The sequencing approach of each eccDNA is also documented on the detail page. We also provide the source literature of each eccDNA, and user can use the link of PMID to obtain the literature in PubMed database (https://pubmed.ncbi.nlm.nih.gov/) (Fig. 4b). Those links enhance the interoperability of data resources and improves the convenience of user retrieval.

#### BLAST

**Fig. 5:**
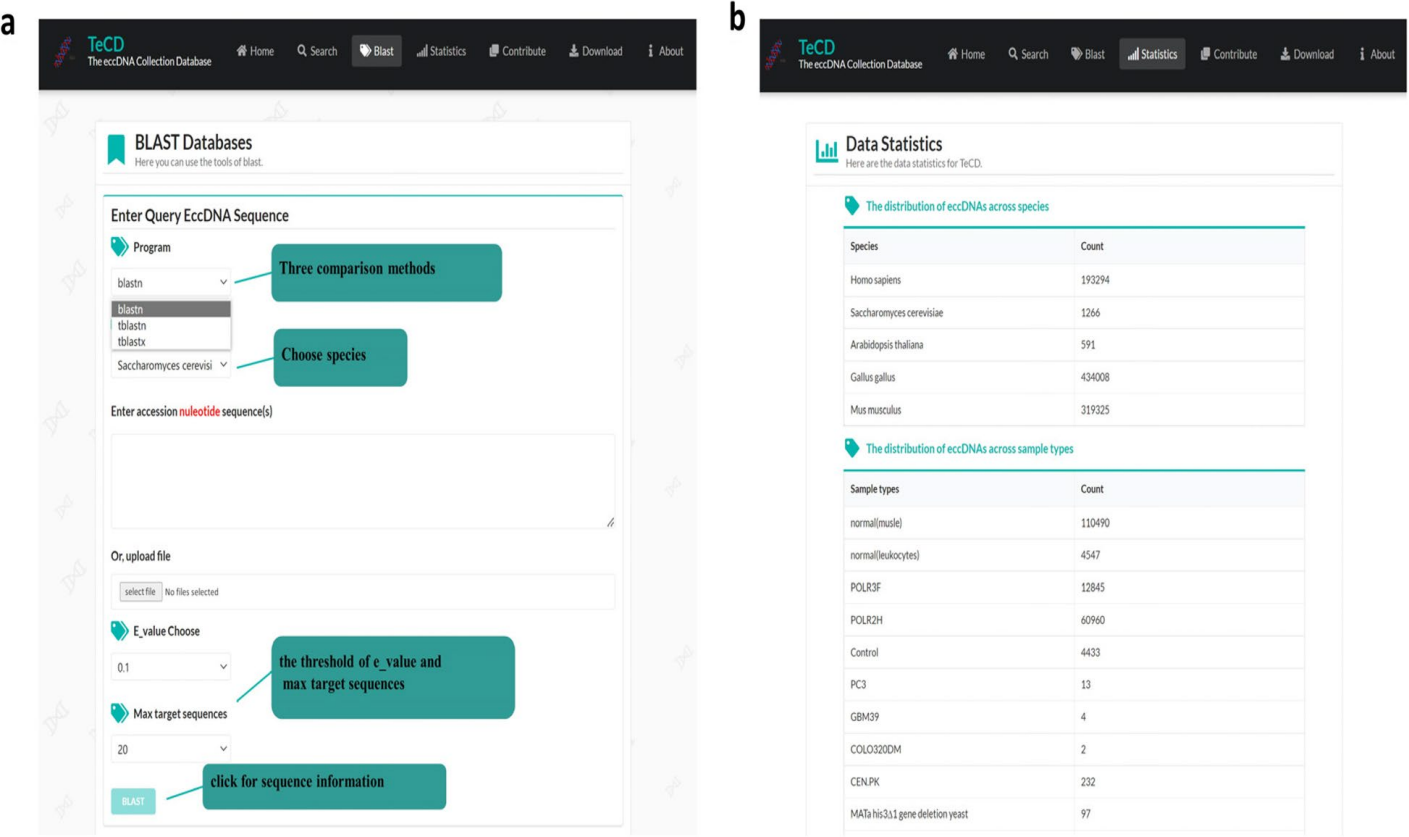
Screenshot of BLAST and statistics page. **a** Screenshot of blast page from the TeCD. Users can use BLAST to search the database by sequence alignment. **b** Screenshot of statistics page from the TeCD

BLAST (basic local alignment search tool) is a set of analysis tools for similarity comparison in protein or DNA databases [42]. There are three BLAST tools to be provided in alignment in TeCD: BLASTN, TBLASTN, and TBLASTX. The BLASTN can compare and aligns an input nucleic acid sequence, and return the most similar nucleic acid sequence in the TeCD [43, 44]. TBLASTN translates all nucleic acid sequences in TeCD into protein sequences and then align an input protein sequence with the translated sequences [45, 46]. TBLASTX can align a translated input nucleic acid sequence with all translated nucleic acid sequences in TeCD and return the most similar sequences [46]. Users can choose the tool according to sequence information and needs (Fig. 5a).

#### Statistics

The statistics page presents the aggregated data in TeCD by tables. It includes the number of genes covered on the eccDNA of each species, percentage of eccDNA with complete gene overlap and partial gene overlap among species, the distribution of eccDNAs across species and sample types. Moreover, the distribution of eccDNAs on chromosomes of each species and the sequencing approach of eccDNAs are also summarized.

#### Contribute, download and about

**Fig. 6:**
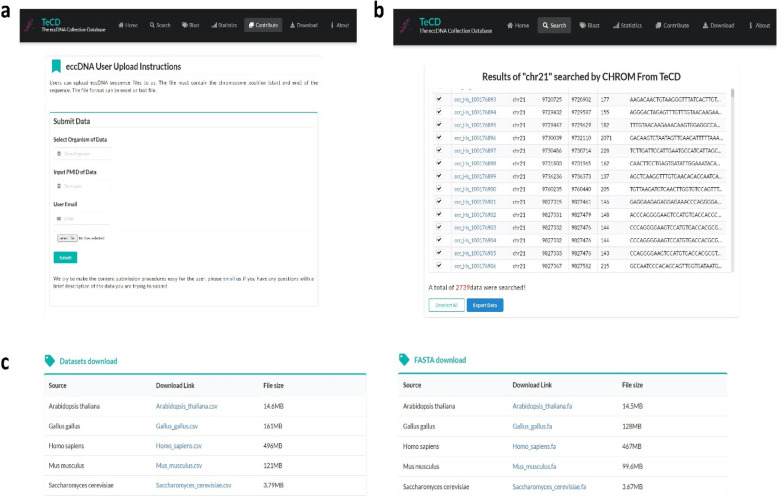
Screenshot of contribute and download page. **a** Screenshot of contribute page from the TeCD. **b** Screenshot of search results page from the TeCD. Users can export some data optionally. **c** Screenshot of download page from the TeCD. TeCD provides download links of all data by species, and the data formats are. csv and. fa

To continuously expand and improve the database, we have built a submission and upload interface, and allow users to upload new eccDNA sequences into the TeCD database (Fig. 6a). We will process the upload eccDNA sequences and integrate them into TeCD in the background.

All data in TeCD are shared under the knowledge sharing license agreement, and we provide a download interface for users to download the data from TeCD (Fig. 6c). Of course, if users want to directly download part of the data they retrieved, they can also check download on the search page. As shown in Fig. 6b, by searching eccDNA data on the human chromosome 21, users can manually select some or all of the data to export. To facilitate users to understand and use our database, we have developed an “About” page to query the meaning of specific items in the detailed sequence information in time. 

### Analyze database sequences using the BLAST

**Fig. 7:**
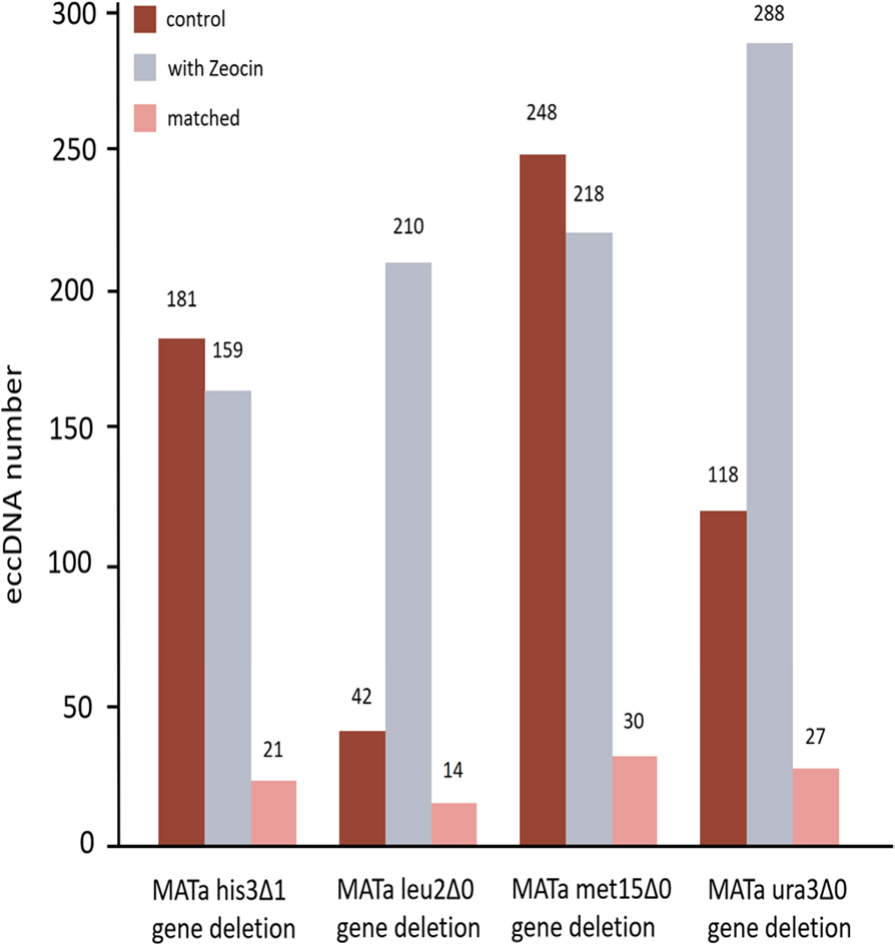
The number of eccDNA with and without DNA damaging. Taking Saccharomyces cerevisiae as an example, a bidirectional comparison of nucleic acid sequences was performed by BLAST

In the Saccharomyces cerevisiae data from the TeCD, there were experimental groups with the DNA damaging agent zeocin and control groups without the DNA damaging agent, to explore the effects of DNA damaging agents on eccDNA. As shown in Fig. 7, the control group had an average of 147 eccDNAs per group (181, 42, 248, and 118 eccDNAs); whereas the group with the DNA damaging agent zeocin had an average of 219 eccDNAs (159, 210, 218, and 288 eccDNAs). Thus, the samples treated with the DNA damaging agent zeocin (219 eccDNAs) showed more eccDNA than untreated samples (147 eccDNAs). We further processed the data and performed bidirectional comparisons of eccDNA sequences obtained from each experimental group and its control group using the platform-built BLAST tool. The result showed that the eccDNA matched by bidirectional comparisons in each group to account for 11%-13% of the average eccDNA in the control and experimental groups. Thus, this result can not only verify that the DNA damaging agent Zeocin can damage the single-strand and double-strand DNA of cells to form eccDNA [23], but also can be used to evaluate the level of DNA strand breakage induced by the DNA damaging agent Zeocin and its effect on eccDNA formation.

## Conclusion

To provide the chromosome source, complete base sequence, and related gene information of eccDNA in different species, we have carried out a large amount of data collection and sorting out, and developed TeCD. We obtained the site information of eccDNA from published literature and matched it with reference genome and gene library data. Thus, we obtained the partially overlapped genes, complete overlapped genes, and transposons. In addition, the sequencing approaches for obtaining eccDNA were also collated and recorded. TeCD web interface allows users to query on-demand and browse the details of each eccDNA. To facilitate users to compare and identify sequences by themselves, we have provided the BLAST function on the web page and all the processed data in TeCD can be downloaded directly by users. TeCD will be updated regularly according to the latest sequencing data and data submitted by users as an interactive website. Based on our knowledge, TeCD is the first database containing the sequence and genomic information of extrachromosomal circular DNA of eukaryotes and BLAST sequence alignment function. Anyway, TeCD will become a powerful tool for researchers to browse and retrieve eccDNA data and further explore the biological functions and contributions of eccDNA.

## Data Availability

All the data are free to use for academic purpose at http://www.eccdna.org:2022 or http://122.224.251.240:2022.

## Abbreviations

EccDNA: Extrachromosomal circular DNA
TeCD: The eccDNA Collection Database
BLAST: Basic Local Alignment Search Tool
NCBI: National Center for Biotechnology Information
ID: Identifier
TAIR: The Arabidopsis Information Resource
HEK: Human Embryonic Kidney
POLR2H: RNA Polymerase II, I And III Subunit H
POLR3F: RNA Polymerase III Subunit F
PC: Prostate Cancer
COLO: Colon Cancer
GBM: Glioblastoma
PMID: PubMed Unique Identifier
